# Multipartner Symbiosis across Biological Domains: Looking at the Eukaryotic Associations from a Microbial Perspective

**DOI:** 10.1128/mSystems.00148-19

**Published:** 2019-06-25

**Authors:** Marta Turon, Maria J. Uriz, Daniel Martin

**Affiliations:** a Centre d’Estudis Avançats de Blanes, CEAB-CSIC, Blanes, Girona, Spain; University of Vienna

**Keywords:** invertebrate-microbe interactions, marine microbiology, symbiosis

## Abstract

Sponges establish tight associations with both micro- and macroorganisms. However, while studies on sponge microbiomes are numerous, nothing is currently known about the microbiomes of sponge-associated polychaetes and their relationships with those of their host sponges. We analyzed the bacterial communities of symbiotic polychaetes (*Haplosyllis* spp.) and their host sponges (Clathria reinwardti, Amphimedon paraviridis, Neofibularia hartmani, and Aaptos suberitoides) to assess the influence of the sponges on the polychaete microbiomes. We identified both eukaryote partners by molecular (16S and COI genes) and morphological features, and we identified their microbial communities by high-throughput sequencing of the 16S rRNA gene (V4 region). We unravel the existence of six *Haplosyllis* species (five likely undescribed) associated at very high densities with the study sponge species in Nha Trang Bay (central Vietnam). A single polychaete species inhabited A. paraviridis and was different from the single species that inhabited A. suberitoides. Conversely, two different polychaete species were found in C. reinwardti and N. hartmani, depending on the two host locations. Regardless of the host sponge, polychaete microbiomes were species specific, which is a widespread feature in marine invertebrates. More than half of the polychaete bacteria were also found in the host sponge microbiome but at contrasting abundances. Thus, the associated polychaetes seemed to be able to select, incorporate, and enrich part of the sponge microbiome, a selection that appears to be polychaete species specific. Moreover, the bacterial diversity is similar in both eukaryotic partners, which additionally confirms the influence of food (host sponge) on the structure of the polychaete microbiome.

**IMPORTANCE** The symbiotic lifestyle represents a fundamental cryptic contribution to the diversity of marine ecosystems. Sponges are ideal targets to improve understanding the symbiotic relationships from evolutionary and ecological points of view, because they are the most ancient metazoans on earth, are ubiquitous in the marine benthos, and establish complex symbiosis with both prokaryotes and animals, which in turn also harbor their own bacterial communities. Here, we study the microbiomes of sponge-polychaete associations and confirm that polychaetes feed on their host sponges. The study worms select and enrich part of the sponge microbiome to shape their own species-specific bacterial communities. Moreover, worm microbiome diversity runs parallel to that of its food host sponge. Considering our results on symbiotic polychaetes and previous studies on fishes and mammals, diet appears to be an important source of bacteria for animals to shape their species-specific microbiomes.

## Abstract (precis)

The symbiotic lifestyle represents a fundamental cryptic contribution to the diversity of marine ecosystems. Sponges are ideal targets to improve understanding the symbiotic relationships from evolutionary and ecological points of view, because they are the most ancient metazoans on earth, are ubiquitous in the marine benthos, and establish complex symbiosis with both prokaryotes and animals, which in turn also harbor their own bacterial communities. Here, we study the microbiomes of sponge-polychaete associations and confirm that polychaetes feed on their host sponges. The study worms select and enrich part of the sponge microbiome to shape their own species-specific bacterial communities. Moreover, worm microbiome diversity runs parallel to that of its food host sponge. Considering our results on symbiotic polychaetes and previous studies on fishes and mammals, diet appears to be an important source of bacteria for animals to shape their species-specific microbiomes.

## INTRODUCTION

Living in symbiosis (in its broader sense) is a general lifestyle across terrestrial and marine ecosystems (1, 2), but it seems to be particularly remarkable in the latter (3–5). Marine sedentary invertebrates, such as sponges and corals, are engineer organisms habitually used as a refuge by diverse mobile fauna. Among them, cnidarians, crustaceans, mollusks, nematodes, and polychaetes are the most frequently reported in association with sponges in temperate, cold, and tropical oceans (6–11).

Many tropical sponges provide refuge to polychaetes. In particular, endosymbiotic species of Syllidae in sponges represent a paradigmatic model for the study of symbiosis, as thousands of individuals of the same worm species colonize one (or a few) sponge species (12–15), and all phases of the polychaete life cycle seem to occur inside the host (13, 16). However, whether these associations are species specific, symbiotic, mutualistic, or parasitic is under discussion (8, 13, 14, 17, 18). While these associations are undoubtedly considered advantageous for the polychaete because sponges represent a food source and a clear refuge against predation (8), the potential benefits for the sponge are more difficult to deduce. Polychaete predation does not seem to cause detectable harm to the host sponges so that the nature of the association has been interpreted as commensalism, mutualism, or “good” parasitism (8, 10, 12, 13, 19).

Indeed, sponge-polychaete associations represent multipartner symbioses as both eukaryotes establish tight associations with multiple microbes (20). Eukaryote partners harbor their own microbiomes, formed of hundreds of bacterial species interacting among themselves and with their respective hosts. Bacteria have been decisive protagonists in the development of the eukaryote cell (21). Since then, they inhabit almost every terrestrial and aquatic niche on our planet and accompany eukaryote organisms along their complete life cycle (22). However, the potential role, if any, of microbiomes in eukaryotic symbiotic associations has not yet been explored. While studies on sponge microbiomes have proliferated in the last decades (23–26), nothing is currently known about the microbiomes of symbiotic polychaetes, including syllids.

In the field of invertebrate-microbe symbioses, how symbiotic bacteria are acquired by a host species remains under debate. Initially, the concept of true symbiont was associated with a maternal inherence (vertically transmitted). Currently, the idea of a species-specific selection of bacteria from the environment by the eukaryote host to form its specific microbiome is gaining support (27–29), particularly since the host’s bacterial composition does not directly reflect that of the environment (28, 30).

Our study identified the bacterial communities of four tropical sponges, *Clathria* (*Thalysias*) *reinwardti* Vosmaer 1880, Amphimedon paraviridis Fromont 1993, Neofibularia hartmani Hooper and Lévi 1993, and Aaptos suberitoides Brøndsted 1934, and those of their respective polychaetes of the genus *Haplosyllis* in different locations of Nha Trang Bay (central Vietnam), aimed at assessing the contribution of the host sponges to the microbiome composition of their associated polychaetes. Considering that syllid worms feed on their host sponges and that diet is known to influence the feeder microbiome, at least in vertebrates (31–34), we hypothesized that polychaete microbiomes would reflect to some extent the microbiomes of their host sponges. In this case, one would expect to find a high degree of similarity between the bacterial communities of the symbiotic partners, with the most abundant members of the sponge microbiome also being major components of the polychaete microbiome.

## RESULTS

### Polychaete identification and associations with host sponges.

**FIG 1:**
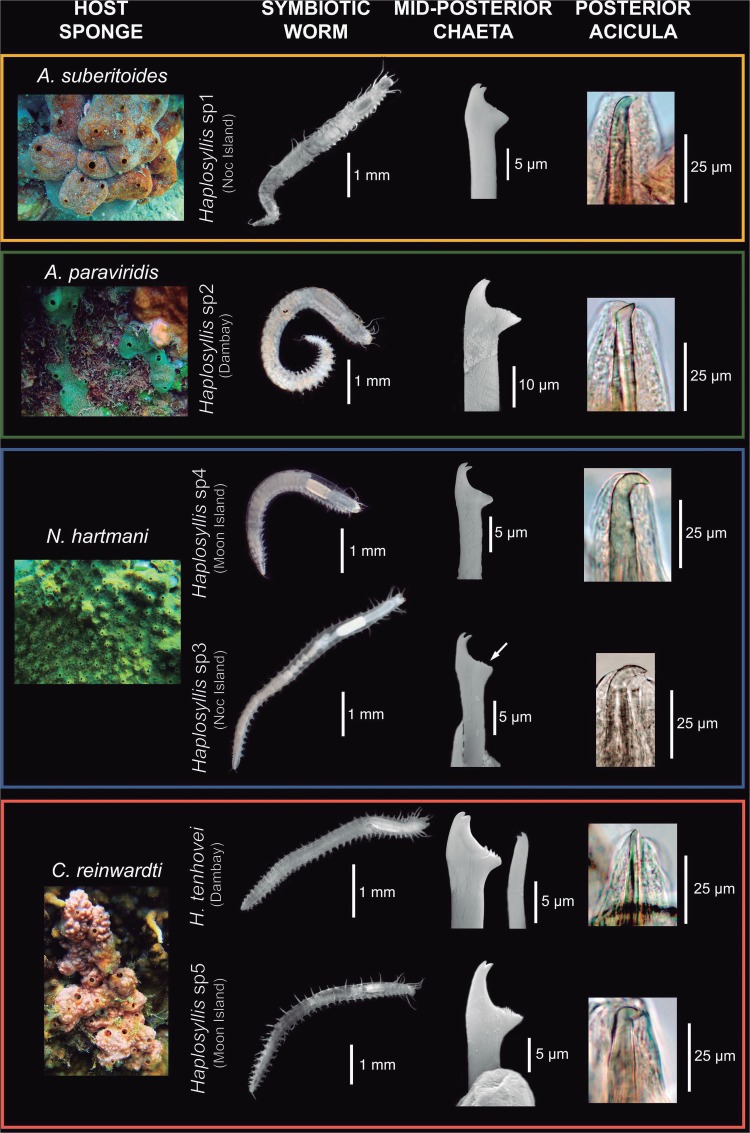
Pictures of the sponges and their associated polychaetes. Scanning electron microscopy photos of the mid-posterior chaetae and optical microscopy photos of the posterior acicula, which were considered diagnostic characteristics for polychaete species differentiation. The locations where the sponges and associated polychaetes were found are indicated in parentheses. Six *Haplosyllis* spp. (five likely undescribed [species 1 {sp1} to 5 {sp5}]) are shown.

All sponge species were dominated by a single polychaete species at high abundance. Figure S1 shows individual worms extracted from a 3-cm^3^ sponge fragment. Six species of *Haplosyllis* could be distinguished based on morphological (Fig. 1) and molecular characteristics. Species identity could be confirmed only for Haplosyllis tenhovei Lattig, Martin, and Aguado 2010, while the remaining five worms likely represented undescribed species, whose formal description will be submitted to a specialized journal and thus, is out of the scope of the present study.

10.1128/mSystems.00148-19.1 FIG S1 Photo of the *Haplosyllis* sp4, individuals extracted from a sponge fragment of *Neofibularia hartmani.* Scale bar is in mm. Photo by Daniel Martin. Download FIG S1, JPG file, 1.5 MB. Copyright © 2019 Turon et al. 2019 Turon et al. This content is distributed under the terms of the Creative Commons Attribution 4.0 International license.

Both 16S and COI sequences (see “Data availability” below for accession numbers) differed among all identified species, except for *Haplosyllis* species 3 (sp3) and *Haplosyllis* sp4, whose sequences are identical despite showing enough morphological differences to be considered different species under traditional taxonomic criteria (Fig. 1).

All respective replicates of Aaptos suberitoides and Amphimedon paraviridis were constantly found in association with a single polychaete species, *Haplosyllis* sp1 and *Haplosyllis* sp2, respectively (Fig. 1). Conversely, in Neofibularia hartmani and Clathria reinwardti, two different polychaete species were found in each sponge, depending on the geographical location. N. hartmani harbored *Haplosyllis* sp3 at Noc Island and *Haplosyllis* sp4 at Hun Moon Island, while C. reinwardti harbored *Haplosyllis* sp5 at Hun Moon Island and *H. tenhovei* at Dam Bay (Fig. 1).

In all cases, evidence of sponge spicules inside the worms confirmed that the symbiotic polychaetes feed on the host sponges (data not shown).

### Sponge and polychaete microbiomes.

**FIG 2:**
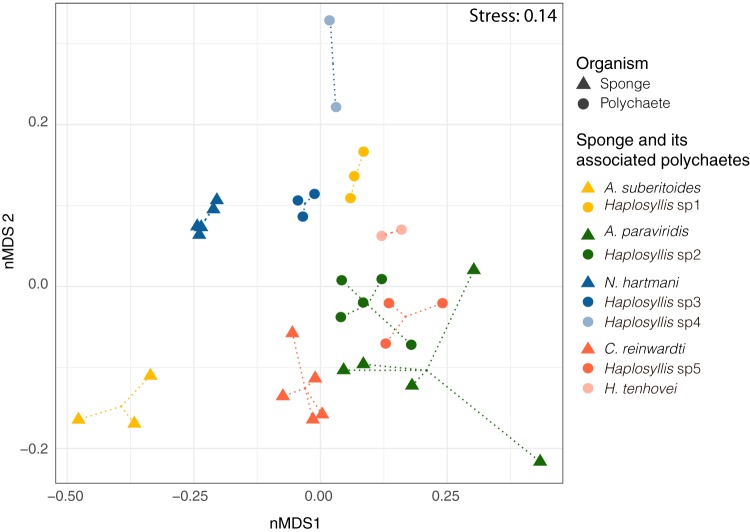
Nonmetric multidimensional scaling (nMDS) ordination of the sponge (triangles) and polychaete (circles) bacterial communities based on Bray-Curtis distances. Sponge species and their associated polychaete species are depicted in the same color.

Host identity was the main factor structuring the bacterial communities of both sponges and polychaetes (Fig. 2) (*R*^2^ = 0.62 and *P* < 0.001 by PERMANOVA [nonparametric permutation analysis of variance]). Polychaete microbiomes had unique bacterial communities markedly different from those of their host sponges and the surrounding seawater (Fig. S2), but they also differed between the worm species.

10.1128/mSystems.00148-19.2 FIG S2 Nonmetric multidimensional scaling (nMDS) ordination of the sponge (green) polychaete (red) and seawater (blue) bacterial communities based on Bray-Curtis distances. Download FIG S2, PDF file, 0.2 MB. Copyright © 2019 Turon et al. 2019 Turon et al. This content is distributed under the terms of the Creative Commons Attribution 4.0 International license.

**FIG 3:**
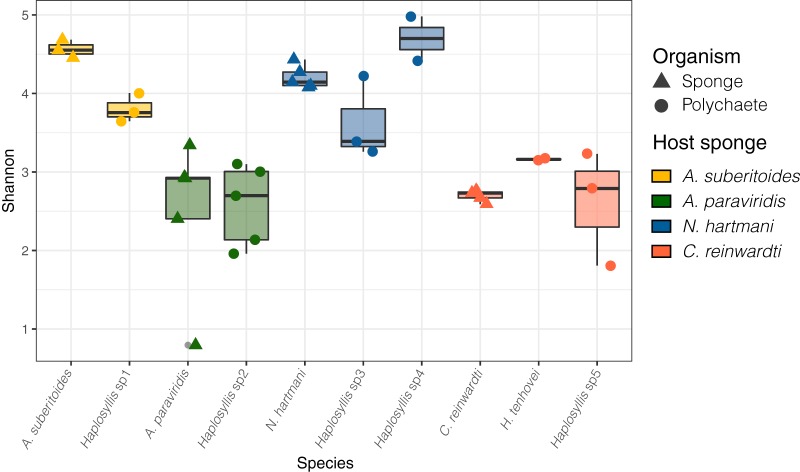
Box plots showing the Shannon diversity of microbiomes in a sponge (triangles) and polychaete (circles). Sponge species and their associated polychaete species are depicted in the same color.

On the basis of Bray-Curtis distances, bacterial communities were more similar to each other in polychaetes than in host sponges (Fig. S3). Although highly different (Bray-Curtis distances > 0.6), microbiome distances in specific associations (host sponge versus its symbiotic polychaete) were significantly lower (*P* < 0.001 by Kruskal-Wallis test) than in nonspecific associations (sponge versus polychaetes from all other sponge species) (Fig. S3). The most similar microbiomes were found in *N. hartmani* and *Haplosyllis* sp3 and in A. paraviridis and *Haplosyllis* sp2 (Fig. S4), while the most distant were those of A. suberitoides and *Haplosyllis* sp1 (Fig. S4). Moreover, the microbiomes of high-microbial-abundance (HMA) sponges (i.e., *A. suberitoides* and *N. hartmani*) were associated with polychaete microbiomes with Shannon diversities higher than the microbiomes of polychaetes associated with the low-microbial-abundance (LMA) sponges (i.e., *C. reinwardti* and *A. paraviridis*) (Fig. 3).

10.1128/mSystems.00148-19.3 FIG S3 Box plot of the Bray-Curtis distances of the bacterial communities between the following: (i) polychaete species, (ii) sponge species, (iv) polychaetes and their host sponge (specific), and (v) polychaetes and nonhost sponges (nonspecific). The specific associations are depicted in green, and the nonspecific associations are depicted in red. Download FIG S3, PDF file, 1.9 MB. Copyright © 2019 Turon et al. 2019 Turon et al. This content is distributed under the terms of the Creative Commons Attribution 4.0 International license.

10.1128/mSystems.00148-19.4 FIG S4 Box plot of the Bray-Curtis distances of the bacterial communities between each polychaete species and its host sponge (specific associations). Polychaete species associated with the same sponge host are depicted in a similar color. Download FIG S4, PDF file, 0.1 MB. Copyright © 2019 Turon et al. 2019 Turon et al. This content is distributed under the terms of the Creative Commons Attribution 4.0 International license.

### Core microbiome communities.

The core bacterial communities of both sponges and polychaetes appeared to be large and represented more than 80% of the relative abundance of the total microbiome in most species (Table S1). A total of 44 ZOTUs (“zero-radius” operational taxonomic units) were detected in all polychaete samples, with the most abundant belonging to *Vibrio*, *Litorimonas*, *Endozoicomonas*, *Pseudoalteromonas*, *Shewanella*, and *Alteromonas* (Table S2). The results shown in the following sections are based on core bacterial communities.

10.1128/mSystems.00148-19.7 TABLE S1 Characteristics of the sponge and polychaeta species. The number of replicates, total number of ZOTUs, number of core ZOTUs, core number of reads reads, and relative abundance of core ZOTUs for each sponge (light gray) and polychaete (darker gray) species are shown. Download Table S1, PDF file, 0.03 MB. Copyright © 2019 Turon et al. 2019 Turon et al. This content is distributed under the terms of the Creative Commons Attribution 4.0 International license.

10.1128/mSystems.00148-19.8 TABLE S2 List of the 44 core ZOTUs present in all polychaete species. The mean relative abundance (as a percentage) and the affiliation at all taxonomic levels are shown for each ZOTU. Download Table S2, PDF file, 0.04 MB. Copyright © 2019 Turon et al. 2019 Turon et al. This content is distributed under the terms of the Creative Commons Attribution 4.0 International license.

### Taxonomic profiles of sponge and polychaete bacterial communities.

The most abundant orders in polychaete communities were *Vibrionales* (24.3%), *Alteromonadales* (17.7%), *Oceanospirillales* (14.3%), *Burkholderiales* (7.6%), and *Caulobacterales* (4.3%), whereas in sponges, they were *Rhodobacterales* (16.29%), *Oceanospirillales* (14.9%), *Nitrosononadales* (8.9%), and PAUC34f unclassified (5.9%).

**FIG 4:**
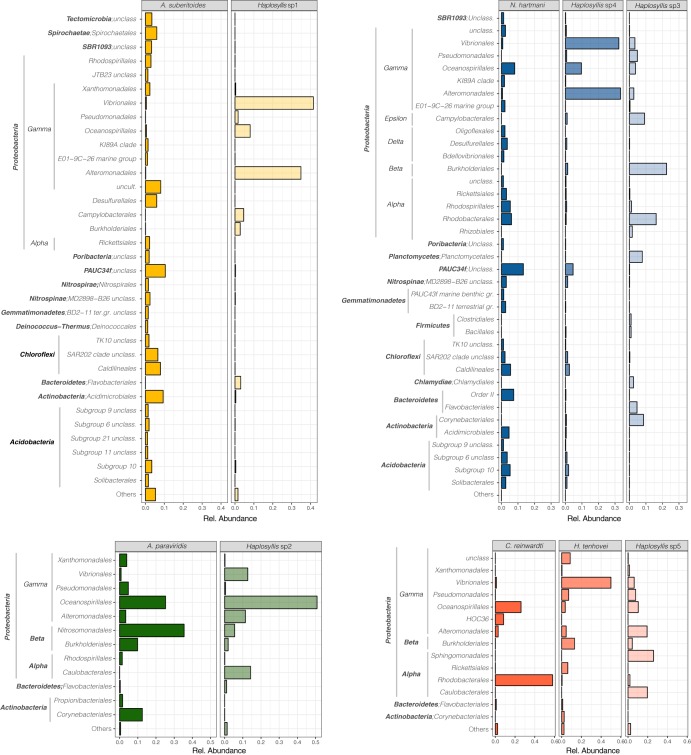
Bacterial composition (at the order level) for each sponge species and its associated polychaetes. The bars represent the relative abundance of each bacterial order in the sponge or polychaete core community. unclass, unclassified; uncult., uncultivated; gr., group.

In most cases, sponges and their associated polychaetes showed highly different bacterial communities (Fig. 4). In *Haplosyllis* sp1, the dominant *Vibrionales* and *Alteromonadales* occurred at relative abundances lower than 0.5% than those in *A. suberitoides*, while in *Haplosyllis* sp2 and *A. paraviridis*, *Oceanospirillales* were highly abundant in both partners. In *N. hartmani* and *C. reinwardti*, each polychaete species (two for each sponge from different localities) inhabiting the same host sponge presented a unique bacterial composition that also differed from the sponge bacterial community. In *N. hartmani*, *Vibrionales* and *Alteromonadales* dominated the microbiome of *Haplosyllis* sp4 (as in *Haplosyllis* sp1 from *A. suberitoides*), whereas *Burkholderiales* and *Rhodobacterales* dominated in *Haplosyllis* sp3. In *C. reinwardti*, *Rhodobacterales* were dominant, whereas *Vibrionales* dominated in H. tenhovei and *Sphingomonadales*, *Caulobacterales*, and *Alteromonadales* dominated in *Haplosyllis* sp5.

### Bacterial communities shared between the eukaryotic partners.

**FIG 5:**
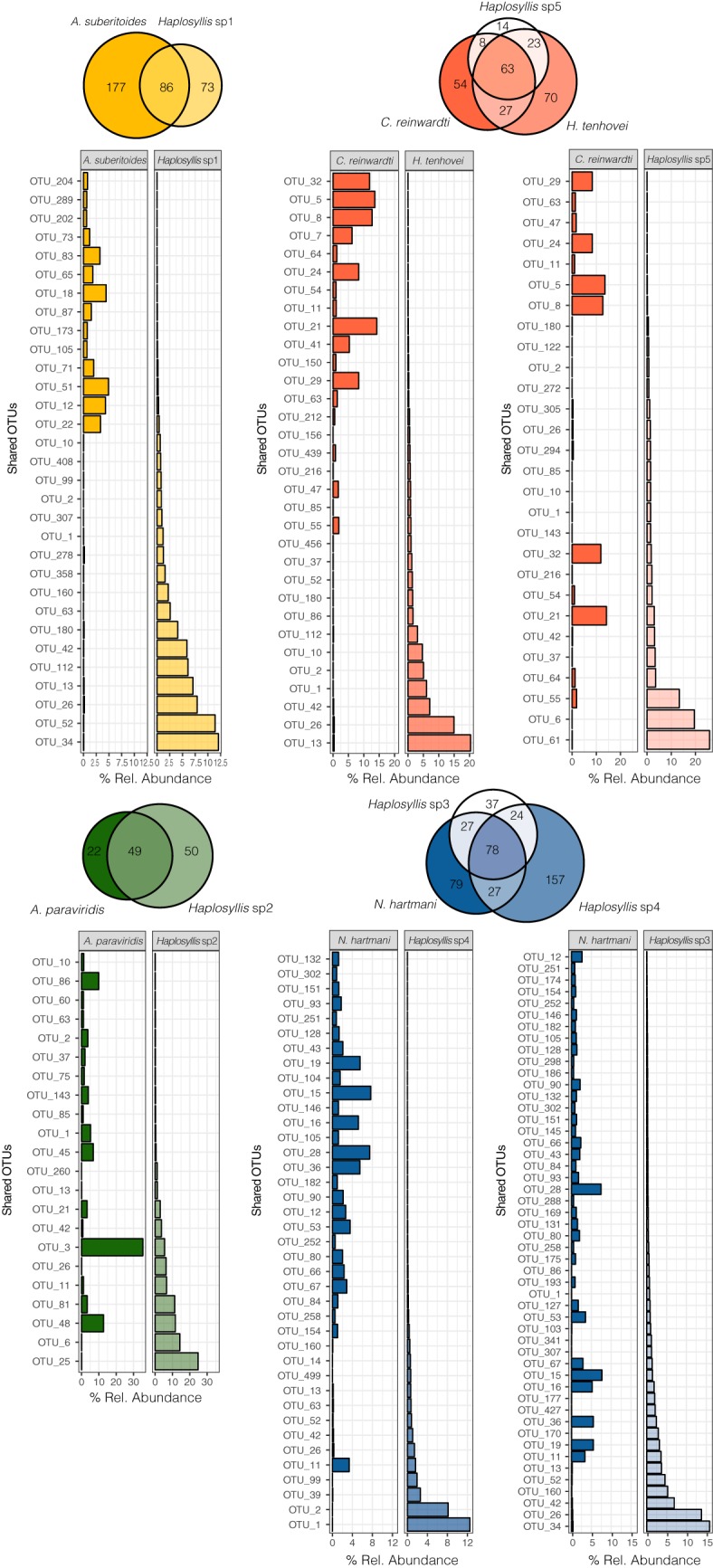
Venn diagrams showing the overlap between the bacterial core communities of each sponge species and its associated polychaete. The size of the circle represents the size of the bacterial core (number of ZOTUs). Bar plots represent the relative abundances (as a percentage) of the shared ZOTUs between each sponge-polychaete system. Only ZOTUs with relative abundances higher than 0.5% in any of the eukaryotic partners are shown in the bar plots.

The number of ZOTUs shared between the sponges and their polychaete symbionts varied among the studied species. More than half of the polychaete ZOTUs were present in their host sponge microbiomes, except for *Haplosyllis* sp4 (Fig. 5), with the most abundant polychaete ZOTUs occurring at low abundances in the respective host sponges and vice versa (Fig. 5). Indeed, the two most abundant ZOTUs of all polychaete microbiomes were found at relative abundances lower than 0.5% in the microbiomes of the respective host sponges (Fig. 5).

Few ZOTUs showed similar relative abundances in both the polychaete and its sponge host (Fig. 5). In the case of *C. reinwardti*, ZOTU 55 belonging to *Shewanella* was also abundant in *H. tenhovei*, and ZOTU 21 (*Endozoicomonas*) and ZOTU 32 (*Rhodobacteraceae*) were both found at high abundances in *Haplosyllis* sp5 ZOTU 48 (*Endozoicomonas*), while ZOTU 81 (*Shewanella*) was abundant in both *A. paraviridis* and its associated polychaete *Haplosyllis* sp2. Finally, in the case of *N. hartmani*, ZOTU 11 (*Endozoicomonas*) was highly abundant in the sponge and in both of its associated polychaetes, and ZOTU 19 (PAUC34f) and ZOTU 36 (*Caldilineaceae* uncultivated) were also abundant in *Haplosyllis* sp3.

**FIG 6:**
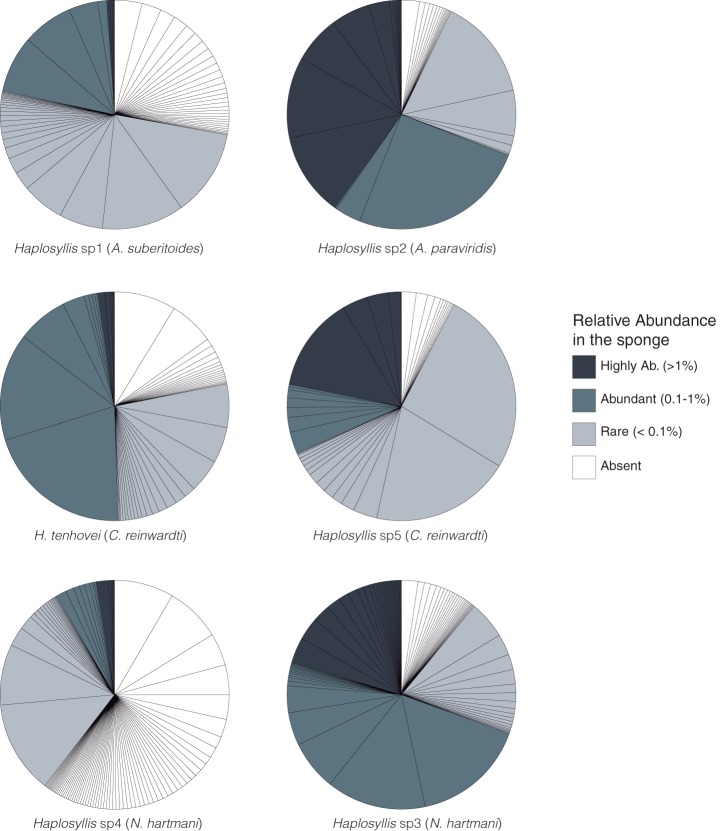
Bacterial core communities of each polychaete species. Only ZOTUs with relative abundances higher than 0.1 in the polychaete core are depicted in the pie charts. Each pie slice corresponds to a polychaete core ZOTU, and its size is proportional to its relative abundance. Colors represent the categorical relative abundance that each polychaete ZOTU is found in the sponge microbiome: highly abundant (dark gray), abundant (gray), rare (light gray), and absent (white).

*Haplosyllis* sp1 (*A. suberitoides*) and *Haplosyllis* sp4 (*N. hartmani*) microbiomes were mainly composed by ZOTUs that were rare or absent in their host sponges (Fig. 6). On the other hand, *Haplosyllis* sp2 (*A. paraviridis*) and *Haplosyllis* sp3 (*N. hartmani*) microbiomes had a greater proportion of ZOTUs that were either relatively abundant or highly relatively abundant in their respective host sponges.

In all cases, polychaetes shared more ZOTUs with the sponges than with the seawater bacterial communities (Fig. S5). Moreover, only a few of the ZOTUs having a relative abundance of >0.05% in the polychaetes were found exclusively in seawater and absent from the sponge (marked with an asterisk in Fig. S6).

10.1128/mSystems.00148-19.5 FIG S5 Venn diagrams showing the overlap between the bacterial core communities of each sponge species, its associated polychaete and seawater. The size of the circle represents the size of the bacterial core (number of ZOTUs). Download FIG S5, EPS file, 2.0 MB. Copyright © 2019 Turon et al. 2019 Turon et al. This content is distributed under the terms of the Creative Commons Attribution 4.0 International license.

10.1128/mSystems.00148-19.6 FIG S6 Bar plots represent the relative abundances (as a percentage) of the shared ZOTUs between each polychaete species, its host sponge, and seawater. Only ZOTUs with relative abundances higher than 0.5% in the polychaete are shown in the bar plots. Download FIG S6, EPS file, 2.5 MB. Copyright © 2019 Turon et al. 2019 Turon et al. This content is distributed under the terms of the Creative Commons Attribution 4.0 International license.

## DISCUSSION

### The sponge-polychaete association.

Observations of tropical worms associated with sponges, most of them classified as Haplosyllis spongicola Grube 1855 have been widely reported (35). However, there is little data available on the relationships between these worms and their host sponges (8, 10, 13). Currently, H. spongicola is known to be a species complex (36) that includes several misidentified species (17), and new species of *Haplosyllis* are continuously discovered. Thus, it was not unexpected that five out of the six species of *Haplosyllis* living in association with the four study sponge species were also undescribed. The Vietnam area seems to be rich in symbiotic polychaetes, according to the numerous species of *Haplosyllis* present there (10, 37), although this can be related to the large number of studies carried out in this area.

We recorded the presence of host-specific spicules in representative samples from all sponge-associated worms. This confirms a sponge-based diet for these symbiotic syllids, as previously proposed for other species (10, 13, 16, 19) and suggests damage to the host. However, the worms’ grazing does not seem to significantly harm their hosts, since they are among the largest and most abundant sponge species in our study area. The absence of negative effects on the hosts would confirm that these associations are commensalistic rather than parasitic, or even mutualistic as recently proposed (10, 19).

On the basis of the study examples, the sponge-polychaete associations appeared to be species specific, that is, all the sponge individuals of the same species in a given area are colonized by the same polychaete species. However, in two cases, the same sponge species harbored two different species of *Haplosyllis* depending on the geographical location. One example is that Haplosyllis nicoleae (instead of *H. tenhovei* in our study) was found associated with *C. reinwardti* in Indonesia (14). On the other hand, ca. 8 of over the more than 30 known symbiotic species of *Haplosyllis* are reported to colonize more than one sponge species (10). Thus, although these associations appear to be species specific at first sight, they may also be ecologically modulated and depend on the geographical/ecological distribution of the species involved. Chemical metabolites released by the host (38, 39) may represent attractant cues for more than one polychaete species (40), so that colonization by one or other might depend on the most prevalent syllid species in a particular area. Colonization by symbiotic polychaetes may be followed by rapid proliferation and complete niche occupation, which could explain the dominance of a single symbiont species in most cases (8, 10, 41).

### Bacterial communities from the eukaryote partners.

Sponge-polychaete symbioses involve many more than two partners, as both eukaryotes harbor particular microbiomes formed by hundreds of bacterial species establishing a tight network of potential interactions. Sponge microbiomes have been intensively investigated during the past 15 years (23–25, 29, 42–44). Conversely, polychaete microbiomes are still poorly known (45–47), with most studies focusing on worms inhabiting hydrothermal vents (48). The microbiomes of the *Haplosyllis* species studied here were more closely related to each other than those of their respective host sponges. Taking into account that all the worms belong to the same genus, while the host sponges belong to different orders (i.e., Suberitida, Poecilosclerida, Desmacellida, and Haplosclerida), we suggest that this pattern may have an evolutionary component.

### Polychaete microbiomes are species specific.

In general, sponge microbiomes tend to be species specific, and the same pattern has been reported for nematodes (49). Our results also show a high species specificity of the polychaete bacterial communities, regardless of their host sponges. Species specificity of microbiomes seems to be more common in invertebrates than previously thought and suggests the existence of species-specific mechanisms of bacterial selection (50), pointing to a relevant role of the associated microbes in invertebrate functioning.

Since polychaete microbiomes appear to be species specific, they may have a diagnostic value in addition to morphological traits. This could be the case of the two species of *Haplosyllis* found in *N. hartmani*, which were morphologically different but molecularly cryptic, and harbor very different bacterial communities. In this sense, microbiomes might inform on ongoing speciation processes even before being detected by molecular markers (e.g., COI and 16S).

### Influence of diet (sponge) on the polychaete microbiomes.

On the basis of previous studies with other organisms, we hypothesized that polychaete microbiomes would reflect those of their prey sponge species. If this were true, two polychaete species feeding on the same sponge would have similar microbiomes. In contrast, *Haplosyllis* sp3 and *Haplosyllis* sp4 feeding on *N. hartmani* and *Haplosyllis* sp5 and *H. tenhovei* feeding on *C. reinwardti* have distinct bacterial communities. Our results suggest that each polychaete species selectively incorporates and enriches specific bacteria, even if these bacteria are rare members of its prey's microbiome. Enrichment of environmentally rare microbes has been reported for sponges (29, 51), mollusks (52), fishes (30, 53), and amphibians (28). Microbiome diversity is positively related between polychaetes and their food source (host sponges), which has also been reported for fish larvae and their food source (34) as well as for the human gut and diet (33). Thus, our results seem to agree with those reported for other organisms, pointing to what could be a widespread pattern relating bacterial diversity of food and feeder. Recently, Cleary et al. (54) also found a compositional similarity between certain sponge samples and sponge denizens, suggesting that sponges may influence the prokaryote composition of organisms that live on or within them or that feed on them.

### Reliance of the polychaete microbiome on the sponge microbiome.

When analyzing the polychaete-sponge relationship from a microbial perspective, we considered that the higher the number of bacterial ZOTUs in the polychaete and absent from the sponge, the lower the polychaete dependence on the sponge microbiome. In this sense, *Haplosyllis* sp1 (from *A. suberitoides*) and *Haplosyllis* sp4 (from *N. hartmani*) would depend less on the sponge microbiome to build up their own microbiome than the remaining polychaete/sponge partnerships studied. The worm bacteria that were not recorded in the host sponge microbiome may possibly correspond to vertically transmitted bacteria (i.e., through sexual or asexual propagula). However, we cannot fully discard some methodological constrains, i.e., if bacteria in the sponge escaped our detection limits. We can also envisage some of these microbes being acquired horizontally from environmental sources other than the host tissues (e.g., from seawater [but see supplemental material]).

In most cases, more than half of the bacteria from a polychaete microbiome, which probably correspond to the gut microbiome, were also found in the sponge, but at contrasting abundances, suggesting different levels of between-partner dependency. It would be interesting to assess to what extent the polychaetes maintain their microbiomes when associated with other sponge hosts with different bacterial communities.

### The polychaete bacterial core.

We have found a quite large core bacterial community in all species of *Haplosyllis*, indicating that polychaete bacteria might play general metabolic or defensive roles (48). Among these core microbes, we found representatives of *Vibrionales*, *Caulobacterales*, *Alteromonadales*, and *Oceanospiralles.* Representatives of these groups have also been reported in other polychaetes such as *Vibrio* in the filter-feeding Sabella spallanzanii Gmelin 1791 (47), *Alteromonadales* and *Oceanospiralles* in deposit feeders Opheliids (45) and *Oceanospiralles* in the bone-eating *Osedax* (55).

Polychaetes have been proposed as bioremediation agents in polluted waters due to their ability to accumulate *Vibrio* species, which are well-known pathogens in aquaculture (47, 56, 57). Conversely, high levels of nonpathogenic *Vibrio* strains have been recently reported in shrimp guts (58), suggesting a possible beneficial role in the invertebrate fitness. Moreover, different members of *Vibrionaceae* are also reported to be extracellular polymeric substances (EPS) producers (59), which are important cell protective agents (i.e., against environmental stressful conditions or from xenobiotic substances) and allow them to capture nutrients (59). In turn, *Alteromonadales* increased in abundance at sites affected by urbanization and eutrophication (45) due to their purported tolerance to high copper levels (45, 60) and to other metals (45, 61). Moreover, members of *Alteromonadales* are well-known EPS and biosurfactant (BS) producers (48, 59), the latter being correlated with antimicrobial activity suggesting a defensive role against pathogens (48). Finally, *Oceanospiralles* are well-known heterotrophic degraders of complex organic compounds (55), which may also contribute to increase the fitness of the associated polychaetes.

### Conclusions.

To summarize, the sponge-polychaete associations seem to be basically species specific but can be ecologically modulated, as different polychaete species inhabited the same sponge species depending on the habitat. The microbiomes of both the sponges and their associated polychaetes are also species specific, pointing to the relevance of the microbial component on the invertebrate functioning. Our results suggest that the associated polychaetes select, incorporate, and enrich a part of the sponge microbiome to form their individual microbiomes, but the selection appears to be species specific, possibly reflecting the specific polychaete needs. Diet appears to be an important source of bacteria for invertebrates (this study) and vertebrates (previous studies) to shape their specific microbiomes.

## MATERIALS AND METHODS

### Sponge and polychaete sampling and DNA extraction.

A quantitative sampling method to describe the sponge assemblages of Nha Trang Bay (central Vietnam) was conducted in April 2015 (29). During that campaign, sponge species associated with polychaetes were surveyed. Four of them were later selected for the present study due to their high abundance and density of associated polychaetes. Among the selected species, *A. suberitoides* and *N. hartmani* belonged to high-microbial-abundance (HMA) sponges, whereas *C. reinwardti* and *A. paraviridis* belonged to low-microbial-abundance (LMA) species (29). Sponges containing polychaetes were collected in April 2016 by SCUBA diving between a depth of 3 and 9 m in three neighboring locations ∼2 km apart (i.e., Dam Bay and Hun Mun and Nock Islands) within Nha Trang Bay. Three samples of *A. suberitoides* (all from Nock Island), five samples of *N. hartmani* (three from Nock Island, two from Hun Mun Island), five samples of *C. reinwardti* (two from Dam Bay Island, three from Hun Mun Island), and five samples of *A. paraviridis* (all from Dam Bay) were collected. Each sponge sample was kept in a 50-ml Falcon tube with native seawater from same depth and sampling point and later replaced by 100% ethanol once the polychaetes left the host sponge (ca. 10 min). The released polychaetes were then cleaned from all remaining sponge tissues and allocated to Eppendorf tubes containing 100% ethanol. Back in the lab, sponges were examined under the microscope to extract any possible remaining polychaetes. In the case of *A. suberitoides*, only a few polychaetes left the host sponge spontaneously, and thus, sponge dissection and careful examination were key to extracting the sponge-associated polychaetes. Ethanol was replaced twice with fresh absolute ethanol to ensure good sample preservation. DNA from sponge and polychaete samples was extracted by following the DNeasy Blood & Tissue kit protocol (Qiagen).

Additionally, triplicate 2-liter water samples were taken from the three locations (ca. 50 cm apart from the sponges) and sequentially filtered throughout 5-μm and 2-μm polycarbonate membranes. The size fraction (5 to 2 μm) was processed for DNA extraction. The membranes were enzymatically digested with lysozyme, proteinase K, and sodium dodecyl sulfate, and afterwards, DNA was extracted with phenol-chloroform-isoamyl alcohol (25:24:1, vol/vol/vol) and chloroform-isoamyl alcohol (24:1, vol/vol). Purification and concentration of the DNA was performed with Amicon Ultra 4 centrifugal filter units with 100,000 nominal molecular weight limit (NMWL) (Millipore).

### Polychaete identification.

Once the polychaetes were separated from their respective host sponges, all polychaetes were carefully identified using a microscope. Anecdotal species (i.e., species other than the most abundant one, present as 1 or 2 specimens per sample) were discarded. Only the dominant symbiotic species from each sponge was considered for this study.

We identified polychaete species to the best possible taxonomic resolution by molecular markers and morphological features. Fragments of the mitochondrial small subunit 16S rRNA gene (∼650 bp) and the cytochrome *c* oxidase subunit I (*COI* ∼680 bp) were amplified and sequenced. Primer pairs 16SarL/16SbrL (62) and jgLCO1490/jgHCO2198 (63) were employed to amplify 16S rRNA and COI, respectively. PCR amplifications were conducted in 50-μl reaction mixtures containing 1 ng of template genomic DNA, 5 μl of 10× PCR buffer (containing 1.5 mM MgCl_2_), 2 μl of dNTP mix (10 mM), 1 μl of each primer (10 mM), and 0.4 μl of *Taq* DNA polymerase (5 U μl^−1^). The temperature profiles to obtain the PCR products were set by following the protocols of Álvarez-Campos et al. (64). Purification and sequencing were conducted by an external service (Macrogen, Spain).

The morphology of the dominant polychaete species, all them belonging to the genus *Haplosyllis*, was observed by using light and scanning electron microscopes following the procedures described by Martin et al. (36). All relevant diagnostic morphological characteristics required for species identification according to Lattig et al. (65) were recorded and then checked against the currently existing literature.

### Verification of polychaete feeding behavior.

From each sponge sample, 25 polychaete specimens were carefully examined to ensure the absence of externally attached sponge spicules, dissolved in boiling nitric acid to totally remove organic matter, and then examined with a light microscope (Leitz Axioplan) to confirm the presence of host sponge spicules in the worm.

### Bacterial 16S rRNA gene amplification, sequencing, and analyzing.

PCR and high-speed multiplexed 16S rRNA gene Illumina MiSeq sequencing (next-generation sequencing [NGS]), were performed following the methods of the genomic core facilities and the methods of MrDNA lab (Shallowater, TX, USA). The variable V4 region of the bacterial 16S rRNA gene was amplified using the primers 564F (5′-AYTGGGYDTAAAGNG-3′) and 785R (5′-TACNVGGGTATCTAATCC-3′) (ca. 250 nucleotides [nt]) (66). Raw rRNA gene sequences were processed separately using the UPARSE pipeline (67). A quality check and dereplication were applied to our data set. Denoising (error correction) of amplicons was performed by using the UNOISE pipeline (68). This algorithm removed chimeras, reads with sequencing errors, PhiX, and low-complexity sequences due to Illumina artefacts, and generates ZOTUs (“zero-radius” operational taxonomic units [OTUs]) with 100% identity sequences.

Taxonomic assignment was done with SINA v1.2.11 (69) using SILVA 128 database. Sequences with low alignment quality (<75%) and sequences identified as mitochondria or chloroplasts were removed from the analysis. To minimize biased effects for differences in sampling effort, the original bacterial ZOTU table was rarefied at a minimum read threshold of 40,000, using QIIME (70). We normalized our data set to the same read count, which means that all data on “bacterial abundance” refer to relative abundance.

### Bacterial community analyses of sponges and their associated polychaetes.

Distance-based multivariate analysis of the sponge and polychaete bacterial communities (at the ZOTU level) was conducted using the *vegan* package in R (71). An nMDS (nonmetric multidimensional scaling) was used to visualize the Bray-Curtis dissimilarity matrix. PERMANOVA (nonparametric permutation analysis of variance), based on 999 permutations as implemented in *adonis* function, was used to test the effect of host identity in the structuring of bacterial communities. We calculated the Bray-Curtis distances between the following microbial communities: (i) polychaete species, (ii) sponge species, (iii) polychaetes and their host sponge (specific), and (iv) polychaetes and nonhost sponges (nonspecific). Shannon diversity (72) of the bacterial communities for each sponge and polychaete species was calculated in *vegan.* The polychaete microbiomes reported here likely reflect the polychaete gut content bacteria more than bacteria from other body regions. However, we were not able to separate the polychaete body regions due to the small body size (>0.5 cm).

Core microbiomes (i.e., ZOTUs present in all species replicates) according to Turon et al. (29) were used for comparing sponge microbiomes with those of their respective polychaete partners. The mean relative abundance of bacterial orders was calculated for each sponge species and its associated polychaete species, and the corresponding Venn diagrams of the shared core microbiomes were drawn using *eulerr* package in R (73). Pie charts were used to represent the relative abundant ZOTUs (>0.1%) in the core communities of each polychaete species and their relative abundance in the core microbiome of the respective sponge hosts, categorized as highly relatively abundant (>1%), relatively abundant (0.1 to 1%), rare (<0.1%), and absent.

Comparisons with seawater bacterial communities were made and are presented as supplemental material. An nMDS was used to visualize the Bray-Curtis dissimilarity matrix of each sponge species, its associated polychaetes, and seawater. The shared microbiomes were represented by using Venn diagrams. The mean relative abundances of shared bacteria between the three biotypes or between polychaetes and seawater were represented as bar plots. Only ZOTUs with a relative abundance of >0.05% in the polychaete microbiome were considered for these comparisons.

### Data availability.

The raw prokaryotic sequences analyzed during the current study are available in the SRA archive under the project number PRJNA453898. Polychaete sequences are available under the GenBank accession numbers MK532398 to MK532403 for the 16S rRNA gene and MK524577 to MK524582 for the COI mitochondrial gene.
