# Supplementary figures and images for: Multipartner Symbiosis across Biological Domains: Looking at the Eukaryotic Associations from a Microbial Perspective
Source: mSystems. 2019 Jun 25;4(4):e00148-19. doi: 10.1128/mSystems.00148-19 (PMC6593219; doi:10.1128/mSystems.00148-19)

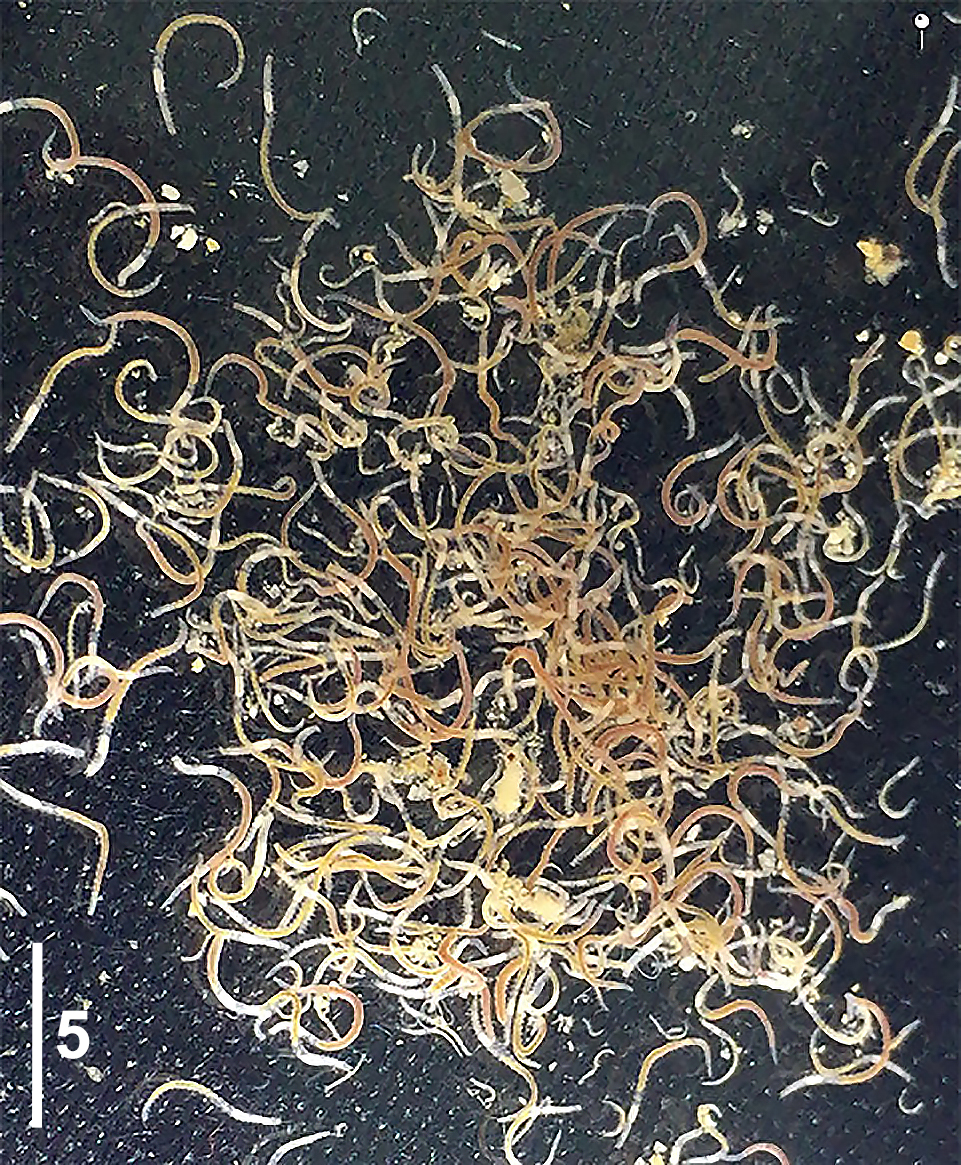

Supplement: FIG S1 [file mSystems.00148-19-sf001.jpg]

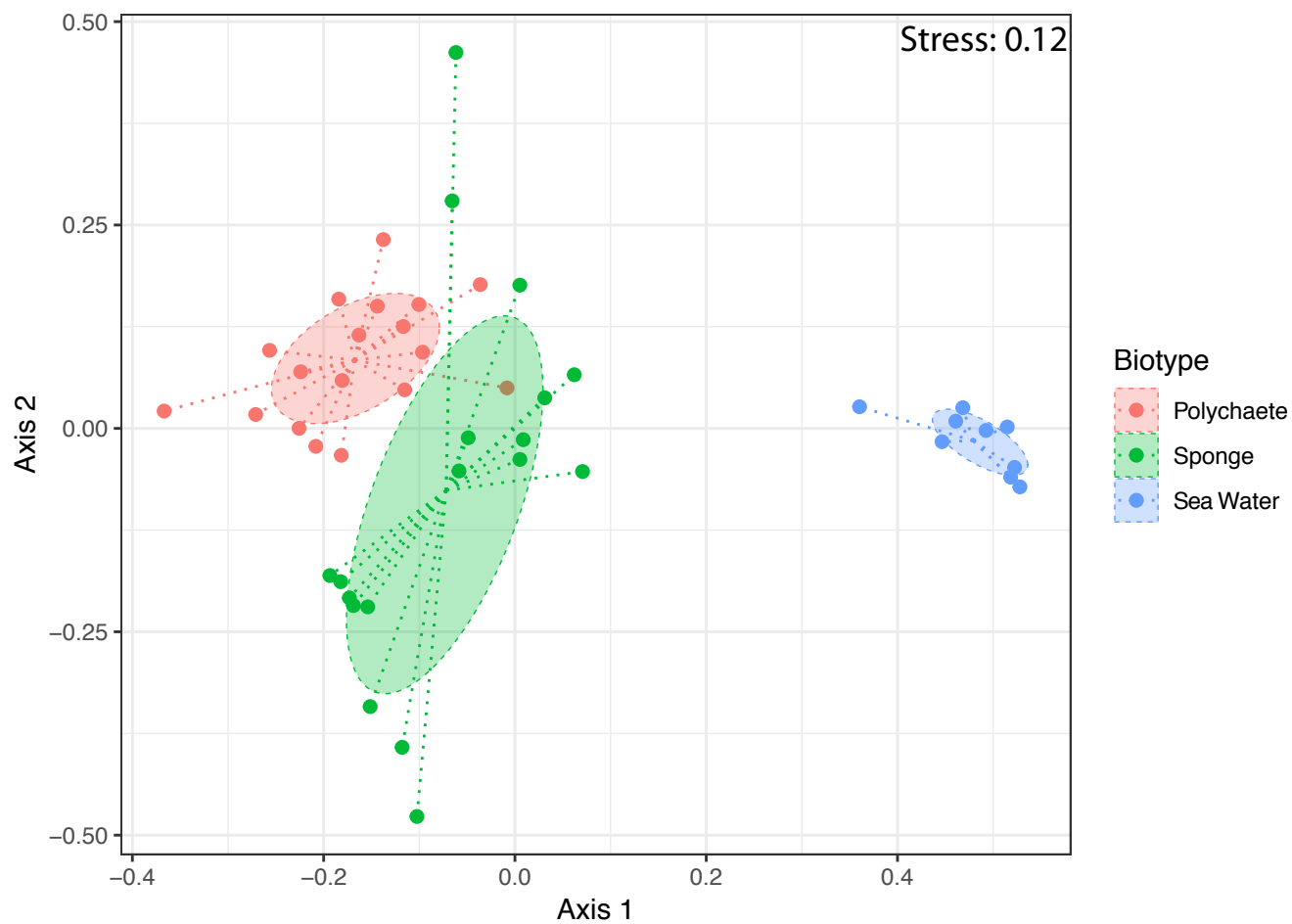

Supplement: FIG S2 [file mSystems.00148-19-sf002.pdf]

Bray-Curtis Disatance

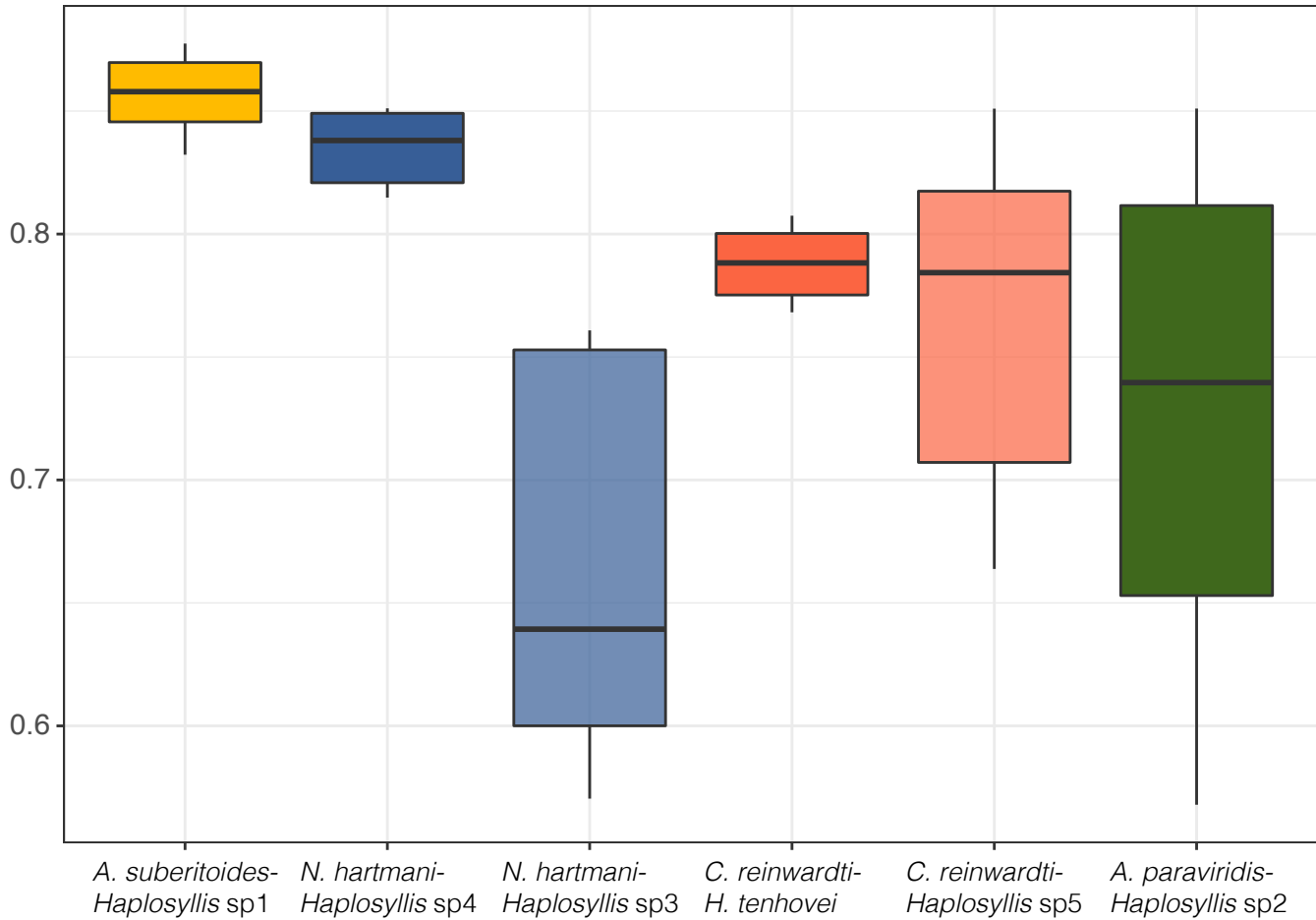

Supplement: FIG S4 [file mSystems.00148-19-sf004.pdf]
