# Supplementary material for: Multipartner Symbiosis across Biological Domains: Looking at the Eukaryotic Associations from a Microbial Perspective
Source: mSystems. 2019 Jun 25;4(4):e00148-19. doi: 10.1128/mSystems.00148-19 (PMC6593219; doi:10.1128/mSystems.00148-19)
Supplement: FIG S3 [file mSystems.00148-19-sf003.pdf]

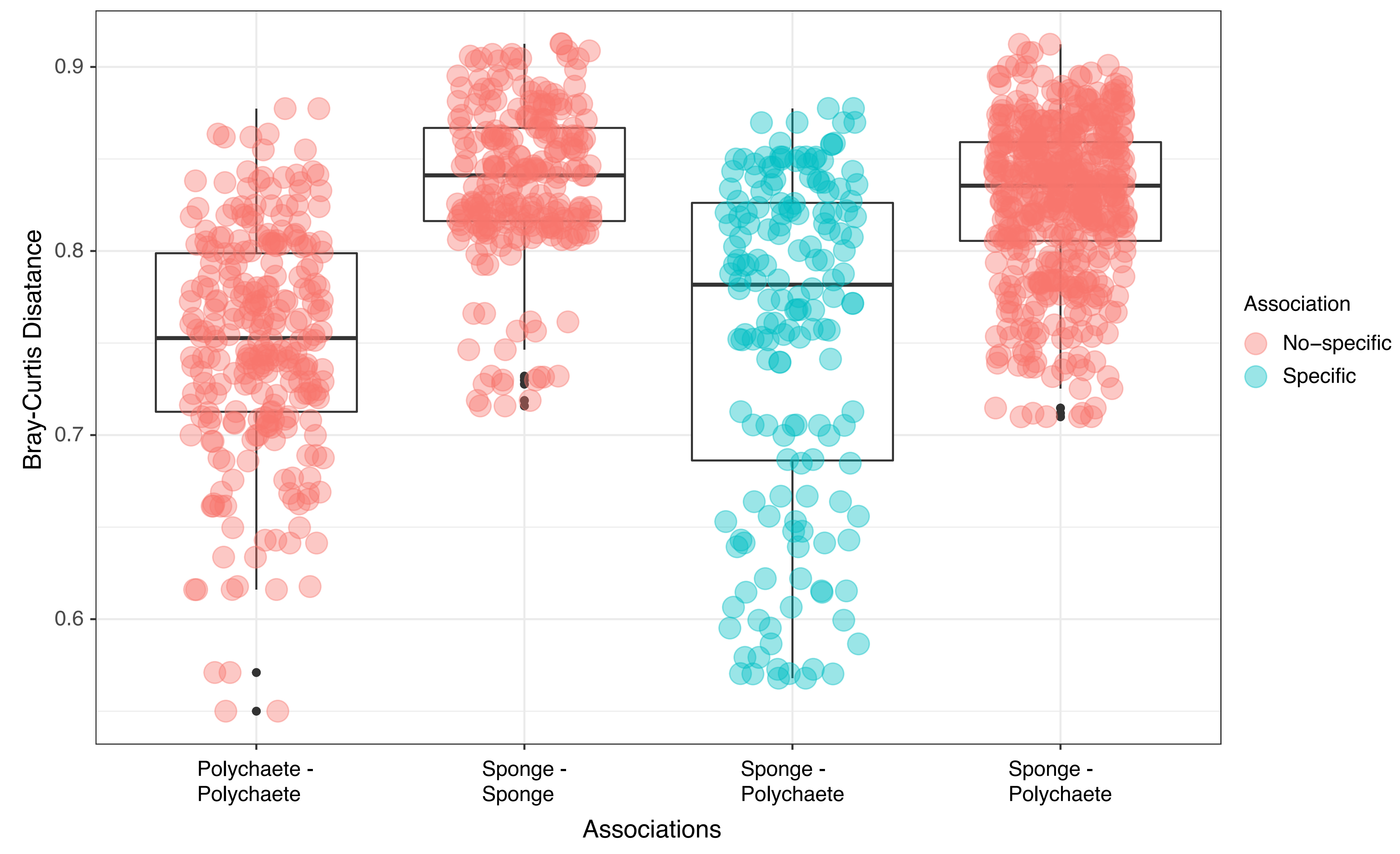

Bray-Curtis Disatance
0.9
0.8
0.7
0.6
Polychaete - Polychaete
Sponge - Sponge
Sponge - Polychaete
Sponge - Polychaete
Associations
Association
No−specific
Specific
