# Supplementary material for: Multipartner Symbiosis across Biological Domains: Looking at the Eukaryotic Associations from a Microbial Perspective
Source: mSystems. 2019 Jun 25;4(4):e00148-19. doi: 10.1128/mSystems.00148-19 (PMC6593219; doi:10.1128/mSystems.00148-19)
Supplement: TABLE S1 [file mSystems.00148-19-st001.pdf]

Species	Num replicates	Num. ZOTUs total	Num. ZOTUS Core	Num. reads Core	Rel. Abundance Core ZOTUS (%)
A. suberitoides	3	1222	263	36196	90.49
A. paraviridis	5	1268	71	21979	54.94
C. reinwardti	5	2525	152	36221	90.55
N. hartmani	5	2096	211	38113	95.28
Haplosyllis sp1	3	1101	159	34138	85.34
Haplosyllis sp2	5	1242	99	35851	89.62
H. tenhovei	2	699	183	35008	87.52
Haplosyllis sp5	3	837	108	32619	81.54
Haplosyllis sp4	2	1343	286	26918	67.29
Haplosyllis sp3	3	985	166	32229	80.57
