# Supplementary material for: Multipartner Symbiosis across Biological Domains: Looking at the Eukaryotic Associations from a Microbial Perspective
Source: mSystems. 2019 Jun 25;4(4):e00148-19. doi: 10.1128/mSystems.00148-19 (PMC6593219; doi:10.1128/mSystems.00148-19)
Supplement: TABLE S2 [file mSystems.00148-19-st002.pdf]

| ZOTU | Mean Relative Abundance (%) | Phylum | Class | Order | Family | Genus |
|---|---|---|---|---|---|---|
| OTU_26 | 6.30 | Proteobacteria | Gammaproteobacteria | Vibrionales | Vibrionaceae | Vibrio |
| OTU_6 | 6.23 | Proteobacteria | Alphaproteobacteria | Caulobacterales | Hyphomonadaceae | Litorimonas |
| OTU_25 | 6.20 | Proteobacteria | Gammaproteobacteria | Oceanospirillales | Hahellaceae | Endozoicomonas |
| OTU_34 | 3.93 | Proteobacteria | Gammaproteobacteria | Alteromonadales | Pseudoalteromonadaceae | Pseudoalteromonas |
| OTU_13 | 3.91 | Proteobacteria | Gammaproteobacteria | Vibrionales | Vibrionaceae | Vibrionaceae-unclass |
| OTU_42 | 3.86 | Proteobacteria | Gammaproteobacteria | Vibrionales | Vibrionaceae | Vibrionaceae-unclass |
| OTU_48 | 2.93 | Proteobacteria | Gammaproteobacteria | Oceanospirillales | Hahellaceae | Endozoicomonas |
| OTU_81 | 2.85 | Proteobacteria | Gammaproteobacteria | Alteromonadales | Shewanellaceae | Shewanella |
| OTU_52 | 2.45 | Proteobacteria | Gammaproteobacteria | Alteromonadales | Alteromonadaceae | Alteromonas |
| OTU_11 | 2.26 | Proteobacteria | Gammaproteobacteria | Oceanospirillales | Hahellaceae | Endozoicomonas |
| OTU_1 | 2.07 | Proteobacteria | Betaproteobacteria | Burkholderiales | Comamonadaceae | Comamonadaceae-unclass |
| OTU_55 | 1.94 | Proteobacteria | Gammaproteobacteria | Alteromonadales | Shewanellaceae | Shewanella |
| OTU_2 | 1.42 | Proteobacteria | Betaproteobacteria | Burkholderiales | Comamonadaceae | Comamonas |
| OTU_3 | 1.39 | Proteobacteria | Betaproteobacteria | Nitrosomonadales | Nitrosomonadaceae | Candidatus Branchiomonas |
| OTU_112 | 1.29 | Proteobacteria | Gammaproteobacteria | Vibrionales | Vibrionaceae | Photobacterium |
| OTU_21 | 1.17 | Proteobacteria | Gammaproteobacteria | Oceanospirillales | Hahellaceae | Endozoicomonas |
| OTU_160 | 1.09 | Proteobacteria | Gammaproteobacteria | Alteromonadales | Pseudoalteromonadaceae | Pseudoalteromonas |
| OTU_10 | 1.02 | Proteobacteria | Gammaproteobacteria | Pseudomonadales | Moraxellaceae | Acinetobacter |
| OTU_180 | 0.93 | Proteobacteria | Gammaproteobacteria | Vibrionales | Vibrionaceae | Photobacterium |
| OTU_170 | 0.79 | Proteobacteria | Gammaproteobacteria | Oceanospirillales | Oceanospirillaceae | Marinomonas |
| OTU_37 | 0.75 | Proteobacteria | Gammaproteobacteria | Pseudomonadales | Pseudomonadaceae | Pseudomonas |
| OTU_63 | 0.52 | Proteobacteria | Gammaproteobacteria | Oceanospirillales | Hahellaceae | Endozoicomonas |
| OTU_64 | 0.49 | Proteobacteria | Gammaproteobacteria | Oceanospirillales | Hahellaceae | Endozoicomonas |
| OTU_85 | 0.41 | Proteobacteria | Betaproteobacteria | Burkholderiales | Comamonadaceae | Delftia |
| OTU_358 | 0.37 | Proteobacteria | Gammaproteobacteria | Vibrionales | Vibrionaceae | Vibrio |
| OTU_143 | 0.37 | Proteobacteria | Gammaproteobacteria | Xanthomonadales | Xanthomonadaceae | Stenotrophomonas |
| OTU_16 | 0.26 | Acidobacteria | Holophagae | Subgroup 10 | TK85 | TK85-unclass |
| OTU_32 | 0.26 | Proteobacteria | Alphaproteobacteria | Rhodobacterales | Rhodobacteraceae | Rhodobacteraceae-unclass |
| OTU_294 | 0.23 | Proteobacteria | Gammaproteobacteria | Alteromonadales | Shewanellaceae | Shewanella |
| OTU_15 | 0.20 | PAUC34f | PAUC34f-unclass | PAUC34f-unclass | PAUC34f-unclass | PAUC34f-unclass |
| OTU_122 | 0.18 | Proteobacteria | Gammaproteobacteria | Enterobacteriales | Enterobacteriaceae | Escherichia-Shigella |
| OTU_14 | 0.14 | Proteobacteria | Betaproteobacteria | Burkholderiales | Comamonadaceae | Comamonas |
| OTU_45 | 0.13 | Proteobacteria | Gammaproteobacteria | Oceanospirillales | Hahellaceae | Endozoicomonas |
| OTU_53 | 0.13 | Proteobacteria | Deltaproteobacteria | Desulfurellales | Desulfurellaceae | G55 |
| OTU_47 | 0.11 | Proteobacteria | Gammaproteobacteria | Oceanospirillales | Hahellaceae | Endozoicomonas |
| OTU_12 | 0.05 | Actinobacteria | Acidimicrobiia | Acidimicrobiales | Sva0996 marine group | Sva0996 marine group-unclass |
| OTU_43 | 0.04 | Proteobacteria | Alphaproteobacteria | Rhodobacterales | Rhodobacteraceae | Albidovulum |
| OTU_31 | 0.04 | Proteobacteria | Betaproteobacteria | Nitrosomonadales | Nitrosomonadaceae | Candidatus Branchiomonas |
| OTU_8 | 0.03 | Proteobacteria | Alphaproteobacteria | Rhodobacterales | Rhodobacteraceae | uncultured |
| OTU_60 | 0.03 | Proteobacteria | Alphaproteobacteria | Rhodospirillales | Rhodospirillaceae | AEGEAN-169 marine group |

| ZOTU | Mean Relative Abundance (%) | Phylum | Class | Order | Family | Genus |
|---|---|---|---|---|---|---|
| OTU_5 | 0.03 | Proteobacteria | Alphaproteobacteria | Rhodobacterales | Rhodobacteraceae | uncultured |
| OTU_33 | 0.01 | Proteobacteria | Deltaproteobacteria | SAR324 clade(Marine group B) | SAR324 clade(Marine group B)-unclass | SAR324 clade(Marine group B)-unclass |
| OTU_115 | 0.01 | Firmicutes | Bacilli | Bacillales | Bacillaceae | Bacillus |
| OTU_118 | 0.01 | Proteobacteria | Alphaproteobacteria | SAR11 clade | Surface 4 | Surface 4-unclass |
